# Safety and efficacy of aneurysms treated with endovascular devices (The SEATED Study)

**DOI:** 10.1093/bjr/tqaf094

**Published:** 2025-04-26

**Authors:** Ahmed R Bassiouny, Anand Sastry, Alex Mortimer, Jeremy Lynch, Hemant Sonwalkar, Aaron Bleakley, Ahmed Iqbal, Ana Paula Narata, Tufail Patankar, Islim Fathallah, Naga Kandasamy, Parthiban Balasundaram, Sara Sciacca, Juveria Siddiqui, Thomas C Booth, SEATED Consortium, Yaman Adi, Peter Atiiga, Paul Burns, Waleed Butt, Arun Chandran, Amar Chotai, Jonathan Downer, Chee Gan, Sergios Gargalas, Changez Jadun, Peter Keston, Kyriakos Lobotesis, Levan Makalanda, Sujit Nair, Marius Poitelea, Prem Rangi, Adam Rennie, Nayyar Saleem, Hannah Stockley, Jonathan Stokes, George Tse, Vicky Young

**Affiliations:** School of Biomedical Engineering & Imaging Sciences, King’s College London, London, SE1 7EH, United Kingdom; Diagnostic Radiology, Faculty of Medicine, Mansoura University, Mansoura, 35516, Egypt; Radiology Department, University Hospital of Wales, Cardiff and Vale University Health Board, Cardiff, Wales, CF14 4XW, United Kingdom; Department of Radiology, North Bristol NHS Trust, Bristol, BS10 5NB, United Kingdom; Department of Interventional and Therapeutic Neuroradiology, Essex Centre for Neurological Sciences, Queen’s University Hospital, Romford, RM7 0AG, United Kingdom; Department of Interventional Neuroradiology, Royal Preston Hospital, Lancashire, PR2 9HT, United Kingdom; Department of Neuroradiology, Royal Preston Hospital, Lancashire, PR2 9HT, United Kingdom; Department of Radiology, Queen Elizabeth University Hospital, Glasgow, G51 4TF, United Kingdom; Department of Neuroradiology, University Hospital of Southampton, Southampton, SO16 6YD, United Kingdom; Interventional Neuroradiology, Leeds Teaching Hospitals NHS Trust, Leeds, LS1 3EX, United Kingdom; Department of Diagnostic and Interventional Radiology, Leeds General Infirmary, Leeds, United Kingdom; Department of Neuroradiology, King’s College Hospital, London, SE5 9RS, United Kingdom; Department of Neuroradiology, King’s College Hospital, London, SE5 9RS, United Kingdom; Department of Neuroradiology, King’s College Hospital, London, SE5 9RS, United Kingdom; Department of Neuroradiology, King’s College Hospital, London, SE5 9RS, United Kingdom; School of Biomedical Engineering & Imaging Sciences, King’s College London, London, SE1 7EH, United Kingdom; Department of Neuroradiology, King’s College Hospital, London, SE5 9RS, United Kingdom

**Keywords:** aneurysms, interventional neuroradiology, endovascular, coils, stents, flow diverters, WEB, safety and efficacy, recurrence predictors, multicenter

## Abstract

**Objectives:**

To create a nationwide consortium to gather all the data related to advanced devices used for aneurysm treatment and conduct pragmatic real-world studies despite the variations among all centres. The strength of this study will be in pooling data of the less commonly used recent devices where there is less evidence. The study will be prospective and retrospective where the initial recruitment figure is expected to be around 5000 patients.

**Methods:**

To assess how endovascular techniques vary among different UK centres, we illustrate the results of initial surveys that were sent to those centres across the United Kingdom using a single device, the pipeline embolization device with vantage technology (PEDV).

**Results:**

Although the centres were using the same device, the antiplatelet protocol varied from one centre to another as well as follow-up protocols according to the local experience, patient clinical status or even according to the adjuncts uses (e.g., adjunct coiling).

**Conclusions:**

The illustrated results show that although the centres were using the same device, the antiplatelet protocol varied from one centre to another. Also, follow-up protocols vary from one centre to another according to the local experience, patient clinical status or even according to the adjuncts used (e.g., adjunct coiling). This exemplar serves to illustrate that a nationwide consortium can pool and analyse data of any recent endovascular device.

**Advances in knowledge:**

Obtaining nationwide data regarding safety, efficacy as well as risk factors for aneurysm recurrence when using recent devices. This study will add valuable data regarding the less commonly used recent devices where there is less evidence.

Ruptured cerebral aneurysms (CAs) account for 85% of non-traumatic subarachnoid haemorrhage (aneurysmal SAH or aSAH). aSAH can be life threatening (with high mortality rate of 44%) and potentially disabling for the surviving groups.^1^ Aneurysms can also go unnoticed, discovered only incidentally on imaging for different indications given that unruptured aneurysms (UAs) are common with a prevalence of 3.2%. Aneurysms tend to occur more frequently and have a higher risk of rupture in females, smokers, older age groups, alcohol and cocaine abuse, those with uncontrolled hypertension and certain genetic medical conditions.^2^ Aneurysms that are larger than 10 mm, located in the posterior circulation, arising from the anterior communicating arterey (ACOM) or the posterior communicating artery (PCOM), or have a daughter sac are more likely to rupture.^3^

Management of aneurysms consists of conservative management or treatment. Treatment is either craniotomy and clipping, or endovascular treatment.^4^

The mainstay of endovascular treatment of aneurysms for over two decades has been coiling which has been used to treat both ruptured and UAs.^5^^,^^6^ However, coils may not be suitable for every aneurysm. For example, wide neck aneurysms can carry the risk of prolapse of coils into the parent vessel which might cause thrombus and ischaemic stroke. These led to the development of balloon-assisted coiling (BAC) as well as stent-assisted coiling (SAC).^7^^,^^8^ Recently developed devices also include intrasaccular devices, which are inserted inside the aneurysm sac, for example, the Woven EndoBridge (WEB) device (Microvention, Aliso Viejo, California, USA)^9^ and Contour neurovascular system (Stryker Neurovascular, Kalamazoo, Michigan, USA).^10^ High mesh-density stents have also been developed as a standalone treatment for aneurysms.^11^ Flow diverters are the name given to these groups of stents that are designed to divert blood flow away from the aneurysm. There are several different flow diverters available, including the pipeline embolization device (PED) (Medtronic, Minneapolis, Minnesota, USA), Surpass (Stryker Neurovascular, Kalamazoo, Michigan, USA), Flow Redirection Endoluminal Device (FRED) (MicroVention, Tustin, California, USA), SILK (Balt, Montmorency, France), and p64 (Phenox GmbH, Bochum, Germany).^12^

There is limited high-level evidence regarding the safety and efficacy of devices. For example, the pivotal international subarachnoid aneurysm trial (ISAT) randomized controlled trial (RCT) comparing safety outcomes after clipping or coiling of ruptured aneurysms, and using Guglielmi detachable coils (GDC),^5^ is now more than 2 decades old and coils, devices, delivery systems, and fluoroscopic equipment have all improved substantially since then. The RCT needed to replicate ISAT for UAs failed to recruit to target. In the decade following ISAT, other RCTs of UA efficacy have been largely limited to endovascular comparisons without including clipping as a comparator. In the last decade, the era of world-leading UK RCTs has receded. Instead, the vast majority of aneurysm treatment studies are not RCTs and are single-arm prospective or retrospective studies from across the world (e.g., stent-coil, flow diverters, intrasaccular). There are a variety of reasons why RCTs have become less common. One reason may be that practitioners are reluctant to randomize, another may be that government funding bodies do not want to fund studies that they think can be funded by industry, and another may be the considerable logistic barriers and costs required to run an RCT, thereby putting it beyond the reach of many funding schemes. Furthermore, industry has led many single-arm studies which are cheaper to run than RCTs and appear to be providing sufficient evidence to allow their devices to be used in current practice.

While high-level evidence is incontrovertibly needed, there is an opportunity for lower-level evidence to be obtained in pragmatic multicentre observational studies at a national level (Box 1). An example is the understanding of outcomes in real-world settings when devices are used outside of limited inclusion criteria from initial studies (Box 2). For example, devices may initially be marketed or approved for certain indications, for example, PED and Food and Drug Administration (FDA) approval for limited cerebral anatomy in USA^13^; or WEB marketed for bifurcations worldwide. Pooling data from these scenarios of extended use contributes to improvements in safety and efficacy in the field of interventional neuroradiology (INR), especially for devices that are used less commonly and have no or little evidence for extended use.

Box 1. A “pragmatic study” is designed to evaluate the real-world effectiveness of an intervention in routine clinical practice. Unlike “explanatory trials,” which focus on determining whether an intervention works under ideal, highly controlled conditions, pragmatic studies aim to understand how well an intervention works in typical healthcare settings with diverse patient populations. There is a spectrum between explanatory and pragmatic studies.

Our pragmatic study would incorporate “off label” use. In the United Kingdom, INRs have the discretion to use a device off-label (ie, for a purpose not specified in its UKCA/CE marking approval) if they believe it is in the best interests of the patient. The UK’s General Medical Council (GMC) states that doctors must:

- Ensure there is strong clinical justification for using a device off-label.
- Consider alternative approved treatments first.
- Obtain informed consent from the patient, explaining the risks and benefits.
- Keep detailed records of the decision and rationale.

Additional relevance of off-label device use relates to cases which result in harm. Legal liability could fall on the individual practitioner (if due diligence was not exercised) and/or the hospital or NHS trust (if use was part of an institutional policy). However, the manufacturer is not liable for any issues arising from off-label use.

Box 2. If in clinical practice, a clinician replicates the exact setting and process of an explanatory trial, similar results and outcomes would be expected. If a clinician does not replicate the trial, the expected results and outcomes would be less clear. No trial can replicate all real-world dilemmas that clinicians face. Therefore, judicious judgement by evidence-informed practitioners is frequently required. This collective judgement of the INR community can be assessed and subsequently re-calibrated through studies such as Safety and Efficacy of Aneurysms Treated with Endovascular Devices (SEATED).

Data mining is the process of discovering patterns, trends, and useful insights from large datasets using statistical, machine learning, and computational techniques. Another opportunity for multicentre observational studies at a national level is therefore to mine data surrounding the determinants contributing to safety and efficacy, for example, the predictors of aneurysm occlusion, alternatively described as risk factors for failure of occlusion. Failure of aneurysm occlusion manifests as recurrence which can be seen on follow-up imaging. Risk factors related to aneurysm recurrences might vary according to the device used and technical factors related to each device (e.g., using a different sized device), or aneurysm factors (e.g., site and size of the aneurysm) or clinical factors (e.g., blood pressure control). If the risk factors are fully understood, it would help reduce recurrences through a modification of risk factors. However, many of these factors are poorly understood particularly for more recent devices. Understanding is compounded by those predictor studies that do attempt to understand risk factors, having methodological limitations. For example, predictor studies analysing the WEB device may be single centre,^14^^,^^15^ or assessed using short-term (6 months) follow-up.^16^^,^^17^ Indeed, this is on the background of there being multiple large WEB studies without a single study being a published RCT. In summary, the opportunity for multicentre pragmatic observational studies of all endovascular devices at a national level will not only contribute to safety and efficacy, especially for devices used less commonly or in extended use, but also will allow determinants of safety and efficacy (e.g., predictors of occlusion etc.) to be better understood. To achieve these broad aims, the Safety and Efficacy of Aneurysms Treated with Endovascular Devices (SEATED) study (HRA IRAS 287395 REC 25/WA/0008) will run initially over a 10-year period allowing all UK centres to perform analyses prospectively and retrospectively in up to 5000 aneurysm cases. The study gathers a wide range of real-world data on both an intention-to-treat and per protocol basis, including patient demographics and aneurysm risk factors, aneurysm characteristics, treatment details including device type and antiplatelet protocols, follow-up imaging outcomes, and clinical follow-up outcomes including complications. The UK INR community are encouraged to develop sub-studies which investigate a particular device. Once a protocol has been established, including inclusion and exclusion criteria, all sites can respond to an expression of interest. Local site Principal Investigators and the central Chief Investigator will share data among themselves (within the limits of the approved over-arching protocol) to deliver the research outcomes.

Success in achieving these broad aims through SEATED is possible because endovascular treatment of aneurysms generates a rich resource of imaging data during follow-up. This is in contrast to many UK non-radiological surgical specialties including neurosurgery where there is often limited follow-up. While it is acknowledged that there is little evidence as to whether extensive imaging follow-up is effective in terms of reducing morbidity and mortality, an important sequela is that interventional neuroradiologists typically have contact with patients for around 4 years after treatment which allows for some limited clinical contact and some limited clinical outcome (safety) data.

A recent UK multicentre observational pragmatic study which was the catalyst for SEATED was a safety and efficacy outcome study of the pipeline embolization device with vantage technology (PEDV) and involved a third of UK sites. Not only was safety and efficacy quantified in a pragmatic setting, but additional real-world insights were inevitably seen for the first time that will influence UK practice.^18^ For example, PEDVU(R) showed that short-term imaging follow-up for UAs using PEDV was performed using DSA in 12.6%, MRA in 84.7%, and CTA in 8%, or a combination of these modalities. The pooled data showed that MRA was considered suitable for the estimation of accurate occlusion rates alongside DSA, but not considered accurate for estimating in-stent stenosis due to susceptibility artifact from the metallic components of the device.^18^ Yet in older studies analysing previous generation PEDs, there had been only limited susceptibility due to different metals used in the device and so MRA was a suitable follow-up modality. These findings show that pooled real-world data are different to data from initial industry-sponsored single (or multi) centre studies where new devices are assessed with intra-arterial (IA) DSA (which is considered by some to be the reference standard for aneurysm follow-up). PEDVU(R) also showed that an effective real-word solution to overcoming the limitations of MRA was to use intravenous (IV) DSA which is less invasive than IA DSA.

We also show additional data to that published in PEDVU(R) demonstrating how UK practice has developed following initial industry-sponsored studies with limited protocols (Table 1). The results show that although the centres were using the same device, the antiplatelet protocol varied from one centre to another. It can also be seen that follow-up protocols varied from one centre to another, and in some centres, this was guided by the adjuncts uses (e.g., adjunctive coiling).

**Table 1:** Results of initial survey to centres using PEDV.

Institution	1	2	3	4	5	6	7	8
**Was platelet resistance testing done?**	No	Yes, only when prasugrel was used	No	Yes	Yes	No	Yes	No
**Antiplatelets used procedural (Elective cases)**	Aspirin 75 mg + clopidogrel 75 mg PO OD for 7 days	Aspirin 75 mg + clopidogrel 75 mg PO OD for 7 days	Aspirin 75 mg + clopidogrel 75 mg PO OD for 7 days	Aspirin 75-500 mg + prasugrel 20-30 mg ≥2 hours prior to procedure	Aspirin 75-500 mg + prasugrel 20-30 mg ≥2 hours prior to procedure	Loading dose aspirin 150 mg + clopidogrel 300 mg 1 week before procedure then 75 mg PO OD for a week before procedure	Aspirin 75 mg + prasugrel 5-10 mg PO OD for 5-7 days	Aspirin 75 mg + prasugrel 5-10 mg PO OD for 5-7 days
**Antiplatelets used procedural (Acutely ruptured cases)**	None was reported	Aspirin 500 mg + prasugrel 20-30 mg ≥2 hours prior to procedure	Loading dose of IV tirofiban bolus and continuous infusion, IV aspirin 500 mg + clopidogrel 600 mg PO OD	None was reported	Aspirin 500 mg + prasugrel 20-30 mg ≥2 hours prior to procedure	Loading dose prasugrel 30 mg 2 hours before procedure + aspirin 500 mg IV at start of procedure	None was reported	None was reported
**Antiplatelets used post procedural (Elective cases)**	Aspirin 75 mg PO OD for life + clopidogrel 75 mg PO OD for 5 to 6 months	Aspirin 75 mg PO OD for life + clopidogrel 75 mg PO OD for 5 to 6 months	Aspirin 75 mg PO OD for life + clopidogrel 75 mg PO OD for 5 to 6 months	Aspirin 75 mg PO OD for life + clopidogrel 75 mg PO OD for 5 to 6 months	Aspirin 75 mg PO OD for life + prasugrel 5 mg PO OD for 3 to 6 months	Aspirin 75 mg PO OD for life + clopidogrel 75 mg PO OD for 5 to 6 months	Aspirin 75 mg PO OD for life + prasugrel 5 mg PO OD for 3 to 6 months	Aspirin 75 mg PO OD for life + prasugrel 5 mg PO OD for 3 to 6 months
	Aspirin 75 mg PO OD for 10 years + clopidogrel 75 mg PO OD for 9 months	Aspirin 75 mg PO OD for life + prasugrel 5 mg PO OD for 3 to 6 months						
**Antiplatelets used post procedural (Acutely Ruptured cases)**				Aspirin 75 mg PO OD for 1 year, occasionally for life				
**Routine follow up Protocol**	MRA-TOF: 6 months MRA-TOF: 18 months MRA-TOF: 48 months DSA: 6 months	MRA-TOF: 6 months MRA-TOF: 24 months	No Coils CTA: 6 monthsCTA: 18 months CTA: 60 months Coils MRA-TOF: 6 months MRA-TOF: 18 months MRA-TOF: 60 months DSA: If indicated	MRA-TOF / DSA: 6 months MRA-TOF / DSA: 24 months MRA-TOF / DSA: 60 months	CEMRA: 6 months CEMRA: 24 months	MRA-TOF: 6 months MRA-TOF: 24 months	No coils CTA: 6 months DSA: 24 months MRA-TOF: 12 months Coils: DSA at 6 months, If DSA shows complete exclusion: MRA-TOF at 24 months. Otherwise: DSA: 6-12 months	MRA-TOF: 6 months MRA-TOF: 18 months MRA-TOF: 24 months MRA-TOF: 36 months MRA-TOF: 60 months
**MRA Machine Details**	- SIGNA 1.5 T HDx, (GE, Boston, Massachusetts, USA) - MAGNETOM Aera 1.5 T, (Siemens, Munich, Germany)	Ingenia 3 T (Philips Healthcare, Amsterdam, Netherlands)	SIGNA Premier 3 T (GE)	SIGNA Premier 3 T (GE)	MAGNETOM Vida 3 T (Siemens)	MAGNETOM Sola 1.5 T (Siemens)	- MAGNETOM Skyra 3 T (Siemens) - Ingenia 1.5 T (Philips Healthcare, Amsterdam, Netherlands)	MAGNETOM Aera 1.5 T (Siemens)

DSA: Digital Subtraction Angiography, MRA: Magnetic Resonance Angiography, TOF: Time-of-Flight, CEMRA: Contrast Enhanced Magnetic Resonance Angiography, CTA: Computerized Tomographic Angiography.

DSA: Digital Subtraction Angiography, MRA: Magnetic Resonance Angiography, TOF: Time-of-Flight, CEMRA: Contrast Enhanced Magnetic Resonance Angiography, CTA: Computerized Tomographic Angiography

We can conclude that real-world data are heterogenous and understanding this is another advantage of SEATED. Objectives include gathering the heterogenous data obtained from various INR practices across the United Kingdom, process and clean these data, and provide results that will play an important role in improving the INR practice (Figure 1). There are multiple research questions where SEATED might contribute and lead to a better understanding of INR practice, including how variability in antiplatelet and follow-up protocols influence aneurysm occlusion rates, and whether there are there significant differences in outcomes between newer and older endovascular devices.

**Figure 1.**
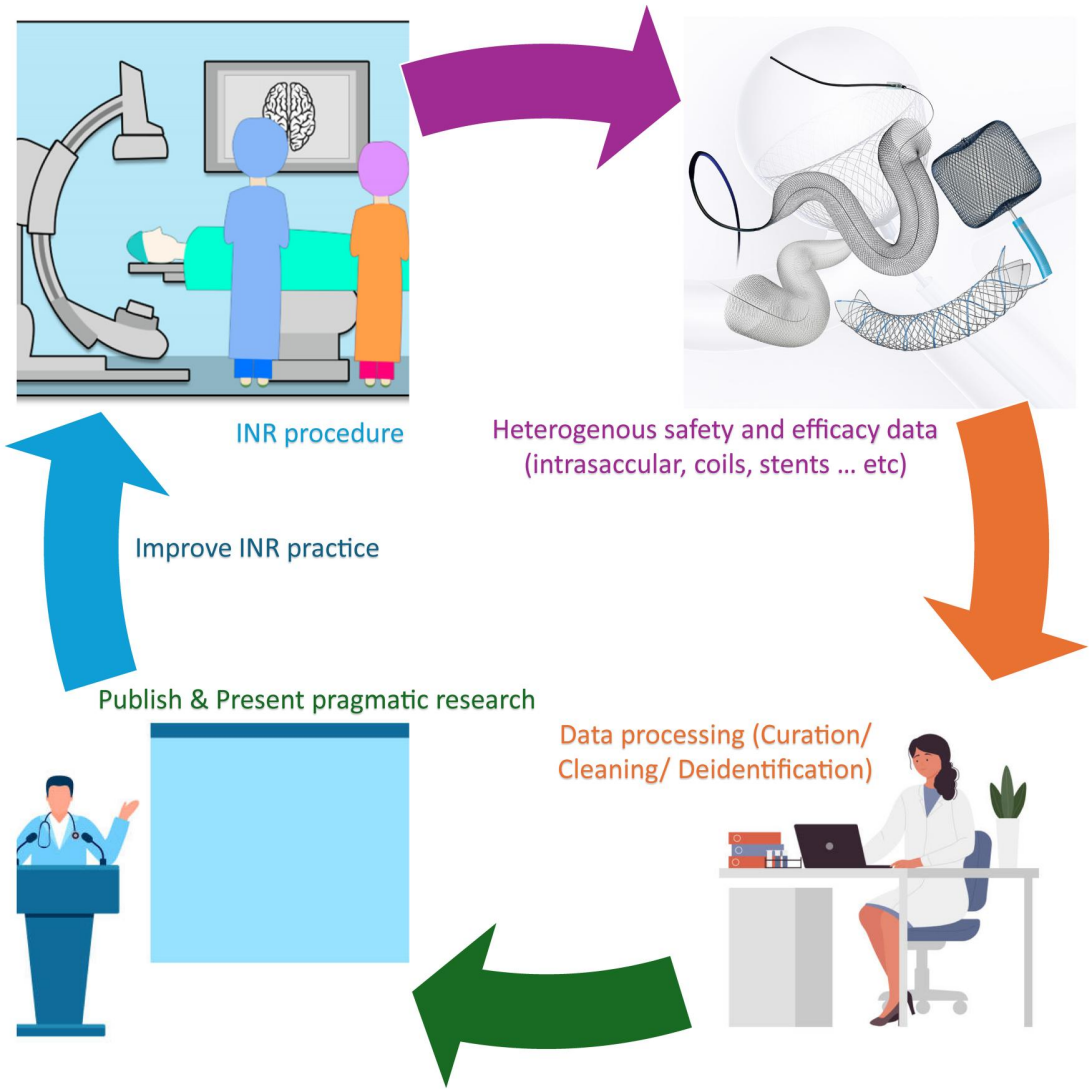
Data gathering process in the SEATED study. Abbreviation: INR = interventional neuroradiology.

SEATED will be prospective and retrospective (like PEDVU(R)), and while the initial recruitment figure is expected to be around 5000 patients, this figure is expected to increase by the end of the recruitment period. The participating centres are illustrated in Figure 2. Two other UK centres have joined provisionally, but currently do not have the capacity to contribute data. Therefore, 93.1% 27/29 UK centres are included representing the majority of UK practice and allowing comprehensive data collection.

**Figure 2.**
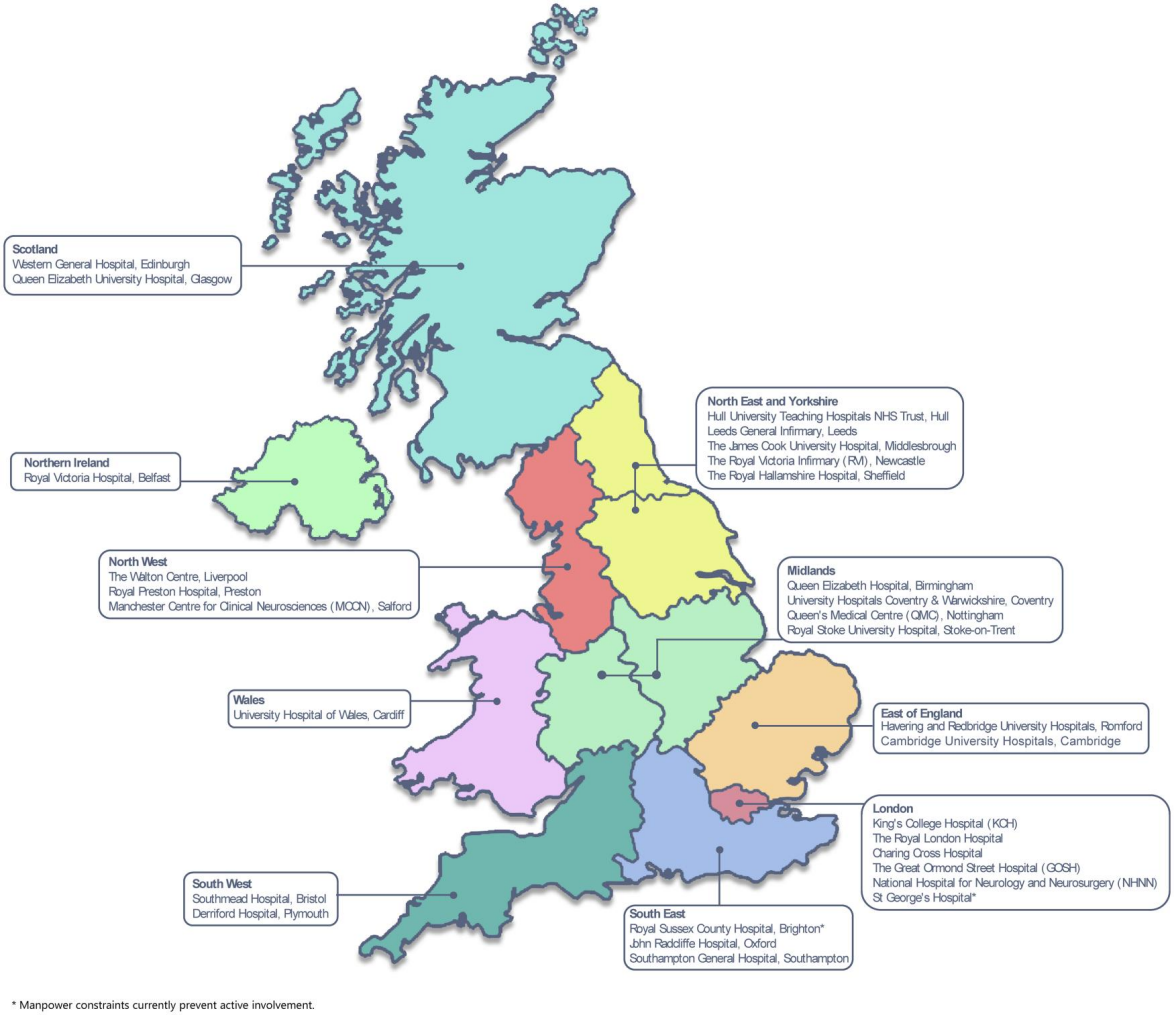
Participating centres in the United Kingdom.

We reiterate that high-level evidence is incontrovertibly needed in addition to SEATED. SEATED is not a replacement for that. However, given that all UK centres are participating in SEATED, with many practitioners contributing to rigorous analysis of practice within their departments, we are already seeing that the network is becoming more receptive to participating in, and leading, high-level evidence studies including RCTs. We also take this opportunity to advocate that the UK neurosurgical community and the INR community must not be reluctant to randomize patients based on weak evidence, or worse still, disincentivized by potential scientific results which would not support their practice. We also advocate that government funding bodies must fund RCTs acknowledging that invariably they are expensive but are the most effective mechanism to be free of bias. Finally, we advocate that industry must fund RCTs as opposed to single-arm studies wherever this is affordable, or where not affordable, support RCTs.

In summary, we are creating a nationwide network to collect data regarding advanced devices for aneurysm treatments to facilitate analysis of safety and efficacy (as primary objectives) and assess factors affecting predictors of safety and efficacy such as aneurysm recurrence after treatment (as a secondary objective). Another secondary objective is far reaching and include determining the best imaging modality for follow-up of particular devices. One particular strength of this study will be in obtaining data for the less commonly used advanced devices where there is less global evidence. Our HRA/REC approved study will run for a decade and allow UK practice to be analysed in depth and ultimately improve patients’ outcomes.
